# Non-revascularized chronic total occlusions impact on substrate and post-ablation results in drug-refractory electrical storm

**DOI:** 10.3389/fcvm.2023.1258373

**Published:** 2023-09-21

**Authors:** Cosmin Cojocaru, Alexandrina Nastasa, Stefan Bogdan, Corneliu Iorgulescu, Alexandru Deaconu, Sebastian Onciul, Radu Vatasescu

**Affiliations:** ^1^ Department of Cardiothoracic Pathology, “Carol Davila” University of Medicine and Pharmacy, Bucharest, Romania; ^2^ Department of Cardiology, Emergency Clinical Hospital Bucharest, Bucharest, Romania; ^3^ Department of Cardiology, Elias University Hospital, Bucharest, Romania

**Keywords:** chronic total occlusion, electrical storm, catheter ablation, risk stratification, ventricular tachycardia, ischemic cardiomyopathy

## Abstract

**Background and aims:**

There is limited data concerning the effect of non-revascularized chronic total occlusions (NR-CTOs) after VT ablation. This study sought to evaluate the impact of NR-CTOs after ablation for electrical storm (ES).

**Methods:**

Post-hoc retrospective analysis of data regarding 64 consecutive post-myocardial infarction patients (out of which 12 patients with NR-CTOs and 52 without NR-CTOs) undergoing substrate ablation for ES with an available median follow-up of 37.53 (7.25–64.65) months. Ablation result was assessed by inducibility of sustained monomorphic VT (SMVT) during final programmed ventricular stimulation (PVS). The primary endpoints were all-cause mortality and VT/VF recurrences after ablation, respectively, stratified by the presence of NR-CTOs. The secondary endpoint was to assess the predictive effect of NR-CTOs on all-cause mortality and VT/VF recurrences in relation to other relevant prognostic factors.

**Results:**

At baseline, the presence of NR-CTOs was associated with higher bipolar BZ-to-total scar ratio (72.4% ± 17.9% vs. 52% ± 37.7%, *p* = 0.022) and more failure to eliminate the clinical VT (25% (3) vs. 0% (0), *p* < 0.001). During follow-up, overall all-cause mortality and recurrences were more frequent in the NR-CTO subgroup (75% (9) vs. 19.2% (10), log rank *p* = 0.003 and 58.3% vs. 23.1% (12), log rank *p* = 0.042 respectively). After adjusting for end-procedural residual SMVT inducibility, NR-CTOs predicted death during follow-up (HR 3.380, *p* = 0.009) however not recurrence (HR 1.986, *p* = 0.154).

**Conclusions:**

NR-CTO patients treated by RFCA for drug-refractory ES demonstrated a higher ratio of BZ-to-total-scar area. In this analysis, NR-CTO was associated with worse acute procedural results and may as well impact long-term outcomes which should be further assessed in larger patient populations.

## 1. Introduction

Non-revascularized chronic total occlusions (NR-CTOs) increase long-term mortality and appropriate implantable cardioverter defibrillator (ICD) therapies in patients with ischemic cardiomyopathy (ICM) (1–4). This has been demonstrated in both primary and secondary sudden cardiac death prevention ICD recipients. Limited data has assessed the prognostic effect of NR-CTOs after ventricular tachycardia (VT) ablation (5–7). However, the impact of NR-CTOs after electrical storm (ES) ablation is unknown.

## 2. Methods

### 2.1. Study population

We performed a single-centre longitudinal retrospective analysis on available data regarding the baseline characteristics and post-procedural course of consecutive patients that fulfilled the following set of inclusion criteria.

- Post-myocardial infarction (post-MI) patients.
- At least three distinct episodes of sustained ventricular monomorphic tachycardia (SMVT) treated by adequate ICD therapies in a 24-h interval refractory to medical treatment and without reversible triggers (8–10).
- Treated by radiofrequency catheter ablation (RFCA) targeting ventricular arrhythmic substrate from January 2014 to June 2021.

Patients with ES induced by acute coronary syndromes, patients with no coronary angiography prior to ablation (two patients) and patients receiving surgical or percutaneous revascularization (one patient during index hospitalization) during the follow-up interval were excluded.

### 2.2. Imaging, electrophysiology study and ablation strategy

All patients underwent coronary angiography during the same hospitalization, prior to the moment of ablation or at the referring hospital, prior to transfer. With the exception of NR-CTOs, potentially significant lesions were defined by ≥70% luminal stenosis (50% for left main lesions), as assessed by two senior interventional cardiologists. Multivessel disease (MVD) was defined by at least one lesion ≥70% simultaneously present or previously treated in at least two epicardial coronary arteries. NR-CTOs were defined angiographically in an untreated [neither surgically nor percutaneously (PCI)] vessel based on the lesion morphology characteristics (as evaluated by two senior interventional cardiologists), irrespective of the degree of anterograde or retrograde collateral flow. NR-CTOs were considered to be incidentally diagnosed by pre-ablation angiography if the patient had no previously documented MI compatible with the NR-CTO localization; NR-CTOs were considered to be clinical if the patient had a previously documented MI compatible to the NR-CTO localization. Mitral regurgitation severity and biplane Simpson-based left ventricular ejection fraction (LVEF) were defined by transthoracic echocardiography formally-recommended criteria prior to ablation (11, 12).

All patients underwent electrophysiological study (EPS) and RFCA in a fasting state under conscious sedation and analgesia. EPS was performed using dedicated recording and analysis system (Boston Scientific Labsystem PRO EP Recording System v.2.7.0.16). High density electroanatomical mapping [>1,800 points, 70% of points focusing on scar and its border-zone (BZ)] was performed in sinus rhythm (SR) with 16–500 Hz signal filtering (CARTO-3™, Biosense Webster, Diamond Bar, California). Mapping/ablation catheter was placed into the RV via transfemoral approach or into the LV via transseptal or retrograde aortic approach. When required, epicardial access was obtained by fluoroscopy-guided anterior percutaneous subxiphoid puncture. Remote magnetic navigation (RMN) (Niobe II, Stereotaxis Inc., St. Louis, MO) and/or multielectrode catheter mapping (decapolar or duodecapolar) was used at the discretion of the electrophysiologist.

Normal myocardium was electrically defined by endocardial bipolar signals amplitude >1.5 mV, LV unipolar signals amplitude >8.3 mV, RV unipolar signals amplitude >5.5 mV and epicardial bipolar signals amplitude >1 mV, while dense scar and borderzone (BZ) myocardium were defined by endocardial bipolar signals <0.5 mv and 0.5–1.5 mV, respectively. Area measurements (total scar area, dense scar area) were manually performed using the integrated CARTO-3 measuring software tool based on the end-procedural voltage electroanatomical map as defined above (BZ scar area and BZ to total scar ratio were derived from the directly measured values). The ablation strategy was based on a previously published scar-dechannelling protocol targeting conduction channel entrances (CCEs) (13) within the scar BZ using open-irrigated ablation catheters (35–50 W, 45°C). Activation/entrainment mapping were performed if VTs were spontaneously or mechanically induced during mapping and were hemodynamically tolerated by the patient.

After elimination of CCEs, a programmed ventricular stimulation (PVS) was routinely performed with at least 2 drive cycle lengths (CLs) and 4 extra stimuli (ESx) [3 ESx in patients with heart failure (HF) symptoms at rest or extreme frailty] (at a minimum of 200 ms or until ventricular refractoriness was reached) from 2 sites of the BZ area (usually medially and laterally to the scar) to assess for VT inducibility [as previously described (14)]. PVS could not be performed in three patients (4.68% of the entire population, all of them without NR-CTOs). If SMVTs [which were considered relevant if their cycle lengths (CLs) ≥250 ms] (15) were induced during PVS, scar reconnection was reassessed and the scar-dechannelling protocol was repeated. If the end-procedural post-ablation PVS induced any SMVT (CL ≥250 ms), the patient was considered to have residual SMVT PVS-inducibility. A SMVT was considered to be the clinical SMVT based on the 12-lead electrocardiogram QRS morphology or based on ICD-derived intracardiac electrograms with similar (±20 ms) CLs. Data regarding procedural characteristics was reported from each patient's last performed ablation procedure.

### 2.3. Follow-up protocol

All patients were monitored from the most recent ES ablation procedure and all the observed events were assessed in relation to the last procedure. Data was obtained from medical records and routine periodical 6 month-interval post-RFCA ICD interrogation. For patients not evaluated in our center, data was obtained from telephone interviews addressed to the referring physicians and patients and from ICD interrogations performed by the referring physicians. ICD interrogation was performed in all patients that were alive in January 2022.

Post-ablation recurrence was defined by SMVT or VF episodes which were adequately treated by either antitachycardia pacing and/or internal electrical shock. Post-ablation detection zones were programmed accordingly to allow detection of any ventricular arrhythmia which was previously spontaneously or PVS-induced (−20 bpm relative the slowest recorded VT). There were no monitoring zones below this VT rate threshold. All-cause mortality rates were retrospectively analyzed during the post-RFCA monitoring interval, irrespective of cardiovascular and non-cardiovascular causes of death.

The study protocol complied with the Declaration of Helsinki and it was approved by the human research committee of the Emergency Clinical Hospital of Bucharest Ethics Committee (12521-01/04/2022).

### 2.4. Endpoints

The primary endpoints were all-cause mortality and VT/VF recurrences, respectively, in the two subgroups (with NR-CTOs and without NR-CTOs, respectively). The secondary endpoint was to evaluate the predictive effect of NR-CTOs on primary endpoints in relation to other relevant prognostic factors.

### 2.5. Statistical analysis

Continuous data was expressed as mean ± standard deviation (SD) for normally distributed data and median (IQR) for non-normally distributed data. Categorical data was expressed as percentage (count). The normality of data was evaluated by Kolmogorov-Smirnov test. Categorical variables were compared using the Fisher's exact test/chi-square analysis and continuous variables were compared using Student t-test if normally distributed and non-parametric tests (Mann-Whitney *U*-Test).

Survival curves were plotted via Kaplan–Meier method and the statistical pairwise over strata comparison between curves was determined using the log-rank test. Univariate and multivariate Cox regression analyses were performed in order to determine the predictive factors. Variables with *P* < 0.2 in the univariate analysis or were then included in the multivariable Cox regression analysis for the determination of hazard ratio (HR) and its 95% confidence interval (CI). The number of predictors assessed in each multivariable model was adapted to the number of events observed during follow-up.

A 2-sided *p*-value < 0.05 was considered statistically significant. Statistical analysis was performed using SPSS version 23 (IBM Corp., Armonk, NY) software and Prism 9 Version 9.5.0 (GraphPad Software, LLC).

## 3. Results

### 3.1. Baseline characteristics

Sixty-four consecutive patients were included and monitored for a median interval of 34.36 (7.25–63.65) months. The baseline characteristics are summarized in Table 1. The median interval between ES diagnosis and the ablation procedure was 0.8 (0.05–2) weeks.

**Table 1:** Baseline characteristics of the electrical storm cohort stratified by the presence of NR-CTO.

	All (*n* = 64)	NR-CTO (*n* = 12)	Without NR-CTO (*n* = 52)	*p*
Males	85.9% (55)	83.3% (10)	86.5% (45)	0.67
Age	62.6 ± 11.1	66.9 ± 7.76	61.6 ± 11.6	0.14
Hypertension	73.4% (47)	75% (9)	73.1% (38)	0.99
T2DM	29.7% (19)	33.3% (4)	28.8% (15)	0.73
CKD	25% (16)	25% (3)	25% (13)	0.99
Active smoker	26.6% (17)	25% (3)	26.9% (14)	0.99
Multivessel CAD	64.1% (41)	83.3% (10)	59.6% (31)	0.18
Single vessel CAD	35.9% (23)	16.7% (2)	40.4% (21)	0.02
Two vessel CAD	31.3% (20	16.7% (2)	34.6% (18)	
Three vessel CAD	32.8% (21)	66.7% (8)	25% (13)	
Prior PCI	59.4% (38)	41.7% (5)	63.5% (33)	0.20
Prior CABG	15.6% (10)	41.7% (5)	9.6% (5)	0.01
Beta-blocker prior to current ES	79.7% (51)	83.3% (10)	78.8% (41)	0.99
Amiodarone prior to current ES	60.9% (39)	66.7% (8)	59.6% (31)	0.75
AF at admission	14.1% (9)	16.7% (2)	13.5% (7)	0.67
NYHA III/IV at admission	29.7% (19)	41.7% (5)	26.9% (14)	0.31
Number of internal shocks	5 (3.5–12)	4.5 (4–6.5)	5 (3–17)	0.35
Clinical VT rate (bpm)	161.36 ± 43.43	156 ± 56.22	162.25 ± 42.38	0.79
Prior CRT	10.9% (7)	0 (0)	13.5% (7)	0.33
LVEF	31.41 ± 10.9	27.7 ± 8.69	32.2 ± 11.3	0.14
Moderate-or-severe FMR	35.9% (23)	66.7% (8)	28.8% (15)	0.02

NR-CTO, non-revascularized chronic total occlusion; T2DM, type 2 diabetes mellitus; AF, atrial fibrillation; NYHA, New York Heart Association; LVEF, left ventricular ejection fraction; FMR, functional mitral regurgitation; VT, ventricular tachycardia; CRT, cardiac resynchronization therapy; PCI, percutaneous coronary intervention; CABG, coronary artery bypass surgery; CAD, coronary artery disease; CKD, chronic kidney disease; ES, electrical storm.

In the NR-CTO group, there were seven (*n* = 7) patients with one NR-CTO and five (*n* = 5) patients with two NR-CTOs. The localization of the NR-CTOs was as following: LAD (41.7%, *n* = 5), LCX (25%, *n* = 3) and RCA (75%, *n* = 9). There were only two patients (16.7%) within the NR-CTO subgroup with incidentally diagnosed NR-CTOs and ten patients (83.3%) with NR-CTOs compatible to the localization of previously documented MIs. Additionally, at the moment of ablation there were five patients with residual potentially significant coronary stenoses [LCX (3.1%, *n* = 2) and RCA (4.7%, *n* = 3)], all of which only received medical treatment during the follow-up interval.

Hospitalization duration was almost two-fold higher in the NR-CTO subgroup (10 (23) vs. 5 (3) days), however without statistical significance (*p* = 0.06).

Overall, 35.9% (*n* = 23) patients had moderate-or-severe functional mitral regurgitation (FMR) (66.7% (*n* = 8) in the NR-CTO subgroup compared with 28.8% (*n *= 15) in the subgroup without NR-CTOs, *p* = 0.02).

### 3.2. Procedural characteristics

Procedural characteristics are summarized in Table 2. The number of procedures per patient were: one procedure (71.9%, *n* = 46), two (20.3%, *n* = 13), three (6.3%, *n* = 4) and one patient (1.6%) underwent four procedures. There were no significant differences between NR-CTO and without NR-CTO subgroups with the exception of failure of clinical VT elimination ablation result which was more frequent in the NR-CTO subgroup compared to those without (25% vs. 0%, *p* < 0.001). Out of the five (*n* = 5) NR-CTO patients with residual SMVT inducibility, three (*n* = 3) developed periprocedural progressive HF symptoms and were deferred from epicardial mapping. The other two patients (*n* = 2) underwent epicardial mapping with no targetable subepicardial substrate.

**Table 2:** Procedural characteristics stratified by the presence of NR-CTO.

	All (*n* = 64)	NR-CTO (*n* = 12)	Without NR-CTO (*n* = 52)	*p*
Multiple procedures	28.1% (18)	50% (6)	23.1% (12)	0.08
Procedure duration (mins)	190.78 ± 69.73	201 ± 60.62	188.23 ± 72.23	0.59
Fluoroscopy duration (mins)	9.36 ± 6.59	7.97 ± 5.63	10.29 ± 7.25	0.43
Duration of hospitalization (days) [median (IQR)]	6 (6)	10 (23)	5 (3)	0.06
Epicardial ablation	10.9% (7)	0 (0)	13.5% (7)	0.33
RMN-guided ablation	76.6% (49)	75% (9)	76.9% (40)	0.99
Multielectrode catheter-mapping during EP study	25% (16)	16.7% (2)	26.9% (14)	0.71
Ablation points	46.41 ± 20.4	46.88 ± 26.04	46.29 ± 19.22	0.91
Total scar area [bipolar, sqcm (SD)]	54.5 (50.3)	74.7 (46)	53.7 (54.8)	0.743
Dense scar area [bipolar. sqcm (SD)]	19.4 (19.7)	20.8 (19.8)	18.4 (24.2)	0.743
Border-zone area [bipolar. sqcm (SD)]	31.2 (32.6)	46 (31.6)	27.7 (32.4)	0.326
BZ to total scar ratio [bipolar. % (SD)]	60.7 (35.8)	72.4 (17.9)	52.0 (37.7)	0.022
BZ to total scar ratio [unipolar, % (SD)]	45.4 (17.7)	54 (17.5)	41.7 (16.7)	0.043
PVS with 4 ESx	68.9 (42)	58.3 (7)	71.4 (35)	0.489
PVS with 3-ESx	31.1 (19)	41.7 (5)	28.6 (14)	
Number of induced VTs	2.14 ± 2.25	1.60 ± 0.84	2.26 ± 2.44	0.40
SMVT at PVS	26.5% (17)	41.6% (5)	23.1% (12)	0.27
Failure of clinical VT elimination	4.6% (3)	25% (3)	0% (0)	<0.001

NR-CTO, non-revascularized chronic total occlusion; VT, ventricular tachycardia; SMVT, sustained monomorphic ventricular tachycardia; PVS, programmed ventricular stimulation; EP, electrophysiological study; RMN, remote magnetic navigation; BZ, border zone.

There were no significant differences in the number of ESx used during PVS in the cases with NR-CTOs vs. those without NR-CTOs (*p* = 0.489). There were three patients from the subgroup with no NR-CTOs which were deferred from post-ablation PVS testing.

The NR-CTO subgroup had a higher bipolar BZ-to-scar ratio (%) compared to the subgroup without NR-CTO (72.4 (17.9) vs. 52.0 (37.7), *p* = 0.022). There were no differences in bipolar BZ-to-scar ratio in the NR-CTO subgroup in the presence of incidental NR-CTOs (71.7% vs. 72.4% in those with localization-compatible MI history, *p* = 0.936). Moderate-or-severe FMR did not influence residual SMVT inducibility at PVS (OR 2.286, CI 95% 0.748–6.986, *p* = 0.147). However, BZ-to-scar ratio did not predict SMVT inducibility (OR 0.985, CI 95% 0.985–1.045, *p* = 0.329).

At final PVS, the NR-CTO subgroup had a significantly higher rate of residual clinical VT inducibility (25% (3) vs. 0% (0), *p* < 0.001) and a higher (yet non-significant) rate of residually inducible SMVTs compared to the subgroup without (41.6% (5) vs. 23.1% (12), *p* = 0.27).

### 3.3. Post-ablation outcomes

**Figure 1:**
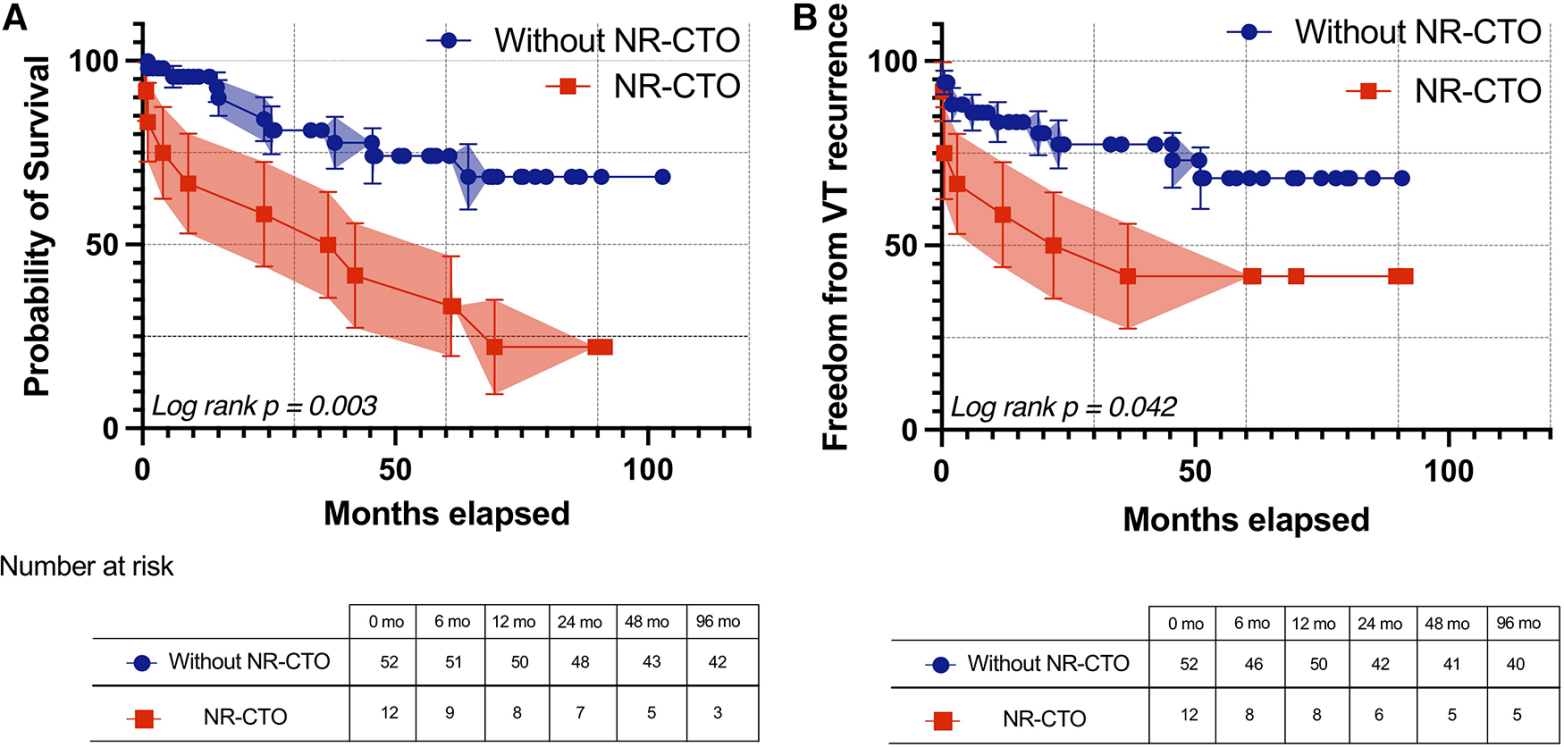
Kaplan-Meier survival curves with 95% confidence intervals of all-cause mortality (**A**) and freedom from ventricular arrhythmia recurrence (**B**) during follow-up in patients with at least one NR-CTO (red line) and without NR-CTOs (blue line). NR-CTO, non-revascularized chronic total occlusion; VT, ventricular tachycardia.

During the follow-up interval, Kaplan-Meier survival curves (Figure 1A) demonstrated that the NR-CTO subgroup had significantly lower survival compared to patients without NR-CTO (log-rank *p* = 0.003) and more frequent recurrences (log rank *p* = 0.042) (Figure 1B).

The overall rate of all-cause mortality during follow-up was 29.7% (*n* = 19). All-cause mortality was higher in patients with inducible SMVT at PVS (55.6%, *n* = 10) vs. those without inducible SMVT at PVS (19.6%, *n* = 9), *p* = 0.007. The overall rate of recurrence during follow-up was 29.7% (*n* = 19). Recurrences were higher in patients with inducible SMVT at PVS (66.7%, *n* = 12) vs. those without inducible SMVT at PVS (15.2%, *n* = 7), *p* < 0.001.

Overall post-ablation, 84.4% *(*n = 54) patients received beta-blockers (NR-CTO 91.7% (*n* = 11) vs. without NR-CTO 82.7% (*n* = 43), *p* = 0.672) and amiodarone in 65.5% (*n* = 42) (NR-CTO 83.3% (*n* = 10) vs. without NR-CTO 61.5% (*n* = 32), *p* = 0.193). Neither post-ablation beta-blockers (*p* = 0.99 for mortality, *p* = 0.99 for recurrences) nor post-ablation amiodarone (*p* = 0.16 for mortality, *p* = 0.56 for recurrences) influenced mortality or recurrences.

### 3.4. Predictive risk factors for clinical endpoints

Cox regression analysis results are summarized in Table 3 and are further detailed in Supplementary Table S1. Residual SMVT at PVS predicted all-cause mortality (HR 5.384, CI 95% 2.155–13.446, *p* < 0.001) and recurrences (HR 7.185, CI 95% 2.666–19.365, *p* < 0.001). In multivariable analysis, residual SMVT at PVS independently predicted all-cause mortality when adjusted by age aHR 4.965, CI 95% 1.847–11.933, *p* < 0.001) and recurrences when adjusted by NYHA III or IV symptoms at admission (aHR 5.214, CI 95% 2.056–13.219, *p* = 0.001).

**Table 3:** Cox regression univariable and multivariable proportional hazards model for prediction of death during follow-up and recurrence during follow-up.

Outcome: Death during follow-up			
Univariable analysis			
Variable	HR	95% CI	*p*
NR-CTO	3.601	1.459–8.886	0.005
NYHA III or IV	2.846	1.148–7.052	0.02
Age	1.093	1.036–1.154	0.001
Residual SMVT	5.384	2.155–13.446	<0.001
LVEF	0.971	0.929–1.013	0.17
Male sex	1.131	0.646–1.980	0.66
Moderate-or-severe FMR	4.299	1.684–10.979	0.002
Multivessel disease	2.242	0.651–7.718	0.201
Multivariable analysis			
Residual SMVT	4.965	1.847–11.933	<0.001
Age	1.102	1.034–1.174	0.003
Outcome: recurrence during follow-up			
Univariable analysis			
Variable	HR	95% CI	*p*
NR-CTO	2.527	0.992–6.435	0.052
NYHA III or IV	2.962	1.198–7.325	0.01
Age	1.042	0.993–1.092	0.09
Residual SMVT	7.185	2.666–19.365	<0.001
LVEF	0.956	0.912–1.003	0.06
Male sex	1.076	0.580–1.995	0.81
Moderate-or-severe FMR	2.122	0.858–5.252	0.10
Multivessel disease	1.139	0.433–2.999	0.792
Multivariable analysis			
Residual SMVT	5.214	2.056–13.219	0.001
NYHA III or IV	2.675	1.059–6.760	0.037

The multivariable models included in this table consist of two of the most significant predictors in univariable Cox regression for death during follow-up and recurrences, respectively (see Supplementary Table S1 for other two-by-two prediction models related to NR-CTO effect and residual SMVT effect on long-term all-cause mortality and recurrences); SMVT, sustained monomorphic ventricular tachycardia; NR-CTO, non-revascularized chronic total occlusion; LVEF, left ventricular ejection fraction; NYHA, New York Heart Association; FMR, functional mitral regurgitation.

Particularly, univariable Cox regression showed that NR-CTO predicted all-cause mortality (HR 3.601, CI 95% 1.459–8.886, *p* = 0.005). In multivariable Cox regression, although NR-CTO remained an independent predictor for all-cause mortality during follow-up when adjusting for residual SMVT at PVS [adjusted HR (aHR) for NR-CTO 5.605, *p* = 0.001], age (aHR for NR-CTO 2.76, *p* = 0.03) and NYHA III or IV symptoms at admission (aHR for NR-CTO 2.09, *p* = 0.003), respectively, it was not significant when adjusting for the presence of moderate-or-severe FMR (*p* = 0.15) (Table 3 and Supplementary Table S1). NR-CTO did not predict recurrence (HR 2.252, CI 95% 0.992–6.435, *p* = 0.052) in univariable Cox regression.

## 4. Discussions

### 4.1. Acute ablation results

In our study, five of twelve NR-CTO patients had residual post-ablation SMVT inducibility compared to the lower rates of positive PVS observed in those without NR-CTOs (23.1%). Moreover, ablation was not able to eliminate the clinical VT in three out of twelve NR-CTO patients (which was however successfully abolished in all those without NR-CTO). This is highly significant as failure to eliminate the clinical VT during ES ablation is strongly associated with short-term very high mortality (16). Furthermore, we emphasize that the PVS protocol was similarly aggressive and there were no significant differences of medical treatment in NR-CTO subjects compared to those without NR-CTOs.

Our results contrast with those of the only three analysed post-VT ablation NR-CTO cohorts in which ablation results were not affected by NR-CTOs (5–7). However, this difference may be influenced firstly by the lower ratio of ES patients included in their analysis (less than one third in the former and ≈60% in the latter) and the relatively lower LVEF observed in our NR-CTO subgroup. Moreover, only three-ESx based PVS was used in Lurz et al.'s protocol which may impact its sensitivity (5).

**Figure 2:**
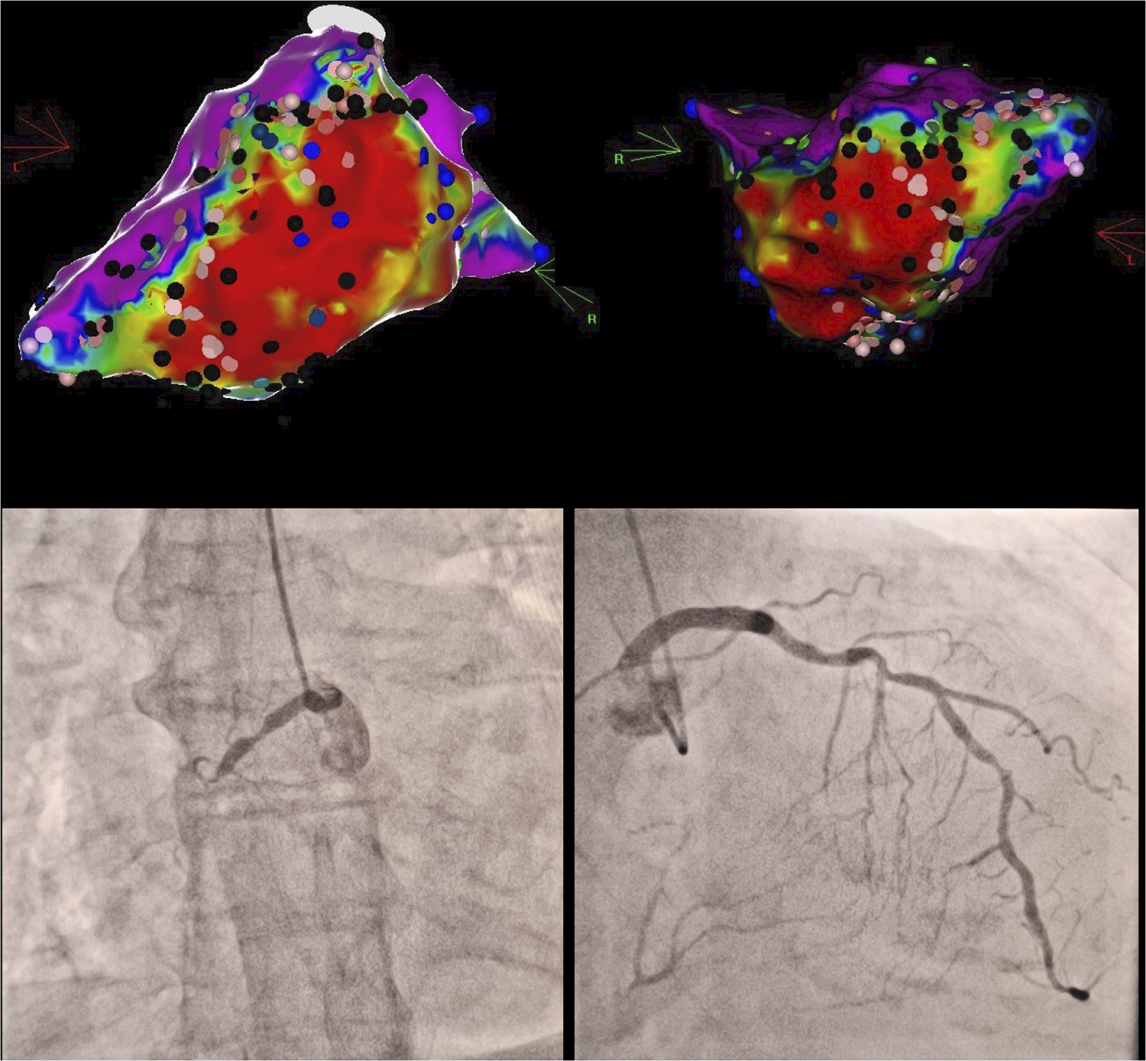
Clinical vignette in a case of proximal RCA chronic total occlusion with contralateral LAD septal collaterals (bottom) and extensive inferior wall to apical segment scar tissue in electroanatomical bipolar voltage mapping (top) with multiple conduction channel entries (black marks), intra-scar late potentials (blue marks).Red areas correspond to dense scar bipolar voltages (<0.5 mV), purple areas correspond to normal bipolar voltages (>1.5 mV), whereas intermediate colours (yellow-green-blue) correspond to border zone myocardial bipolar voltages (0.5–1.5 mV). RCA, right coronary artery; LAD, left anterior descending.

We hypothesize that our observations can be explained by more complex substrate which limits RFCA efficiency, especially in ES acute settings. In the presence of CTOs, scar border-zone (BZ) area is usually expected to be larger and more heterogenous (6) which strongly correlates with both spontaneous incident VTs (17) and VT inducibility at PVS (18). Most myocardial segments which are supplied by NR-CTOs have less than 50% scar transmurality (19). Even more, recanalization of CTOs may promote reverse remodelling particularly within the BZ (20). Hence, the presence of infarct-related CTOs is known to double the risk of ES development (21). Our dataset showed that although the total scar area was not significantly higher in the NR-CTO subgroup (74.7 sqcm vs. 53.7 sqcm), there was however a significantly higher proportion of BZ myocardium within the total scar identified at electroanatomical mapping (72.4% compared to 52% in those without NR-CTOs) (Figure 2). Additionally, we considered the lack of epicardial ablation in the NR-CTO subgroup is most likely a result of the reduced analyzed sample. Out of the five NR-CTO patients with residual SMVT inducibility, three developed periprocedural progressive HF symptoms and were deferred from epicardial mapping. The other two patients underwent epicardial mapping with no targetable subepicardial substrate.

Considering that almost all CTOs generate myocardial ischemia even at rest, as proven by abnormal fractional flow-reserve (FFR), irrespective of collateral flow (22), it may be speculated that variable coronary flow can lead to changes in relevant electrical substrate which hinders its complete characterization during EPS (which can alter ablation results and, importantly, subsequent recurrences).

In addition, NR-CTO patients exhibited significantly more extensive CAD and significantly more frequent moderate or severe FMR. All of these may contribute to the trend of more severe HF symptoms (five out of twelve NYHA III/IV) in the NR-CTOs subgroup. Furthermore, more extensive CAD, systolic dysfunction and, interestingly enough, better developed collateralization have all predicted development of ischemic FMR after primary PCI in ST-elevation MIs (23).

Therefore, the presence of NR-CTOs may impact ES ablation outcomes due to substrate complexity and disease severity.

### 4.2. Long-term clinical course after ES ablation

In our cohort, post-ablation ES patients demonstrated high all-cause mortality (29.7% overall & 9.4% in the first year) and recurrences (29.7% overall & 20.3% in the first year). We observed that the most prominent driver of mortality in univariable analysis (5-fold higher risk) and recurrences (7-fold more probable) is residual SMVT inducibility at post-ablation PVS which is in line with previous publications (5, 6). Furthermore, residual SMVT maintained predictive effects on death and recurrences when adjusted by the presence of NR-CTOs and all other included factors. There are, however, multivariable models which have shown that other predictors (such as disease severity, comorbidities and procedural complications) may outweigh the effect of persistent inducibility at PVS (24).

In the presence of NR-CTOs, overall all-cause mortality (75%) and recurrences (58.3%) were significantly higher. However, these observations stem from a limited number of included patients (particularly with NR-CTOs) and should be further evaluated in larger samples. The excess mortality and incident VT episodes associated with NR-CTOs has been demonstrated in ICM patients, particularly in primary and secondary prevention ICD recipients (1–4, 25, 26). In addition, previous studies suggest that outcomes may be improved after NR-CTO revascularization (27). There is however, very limited published experience with NR-CTO patients after VT ablation (5–7).

Our analysis suggests that NR-CTOs independently predicted death after ES ablation when adjusted by SMVT inducibility at PVS, severe HF symptoms at admission, age and LVEF, respectively. Existing data suggests this effect was attenuated by confounding factors during a shorter interval of monitoring (≤20 months) predominantly after VT ablation (not ES) (5). One possible explanation of this difference could be the significantly longer monitored interval in our study. As previously emphasized, ES development in ICD recipients can be both a cause and an effect of HF progression, especially in certain HFrEF subgroups of patients (28). We believe it is highly valuable to distinguish previously stable patients which are more likely to respond well to ablation as opposed to end-stage HF cases which should rather receive specific advanced HF treatment such as cardiac transplant or mechanical devices.

In our cohort, NR-CTOs did not predict recurrences, which contrasts Di Marco et al.'s observations (6). This was especially evident when adjusting for PVS inducibility, which was not previously included in any prediction models. PVS (especially aggressive protocol with 4-extrastimuli) unmasks residual arrhythmogenic substrate which may become relevant for subsequent VT episodes which may explain why it drives recurrence prediction during follow-up.

Although significant coronary stenoses should be revascularized prior to ES ablation, addressing NR-CTOs before ablation may not be a reasonable option (especially if J-CTO scores are high). However, the NR-CTO effect on long-term mortality suggest that this decision should be revisited after ES treatment, as selective revascularization may improve outcomes, particularly guided by viability (27). Considering the ongoing ischemia attributed to NR-CTO even at rest, tackling HF-inducing mechanisms by targeted therapies (as shown by the anti-arrhythmic effect of CRT vs. ICD in propensity-matched previous registries) may deny the manifestation (or recurrence) of ES or non-clustered VTs (29).

In summary, our data suggests there may be incremental mortality attributable to the presence of NR-CTOs in ablated ES patients. However, it seems not to be driven by recurrences, but by other mechanisms (most likely due to progressive HF deterioration). Consequently, identifying NR-CTOs in ES patients may warrant close monitoring after ablation due to the higher risk of unfavourable outcomes.

Last but not least, advanced age and severe HF symptoms at admission maintained independent prediction of death after ES ablation when adjusted by NR-CTO and positive PVS, respectively. This is in line with previous dedicated prognostic scores such as PAAINESD and I-VT (30–32). Furthermore, moderate-or-severe FMR is known to independently aggravate outcomes in HF patients (25, 33) and attenuated the effect of NR-CTOs on all-cause mortality. In our dataset, moderate-or-severe FMR did not influence recurrences.

## 5. Limitations

1. Data regarding NR-CTOs and CAD complexity were retrospectively acquired on a limited number of patients which were included in a post-hoc analysis. However, our cohort solely consisted of ES patients as opposed to previous cohorts which also included isolated VT cases.
2. The small number of events during follow-up (19 deaths and 19 recurrences) limited the maximum number of variables to be included in multivariable Cox regression models. Two-by-two Cox regression models have been included in Supplementary data section (Supplementary Tables S1 and S2). This may hinder the complete understanding of each factor's effect in ES long-term clinical course.
3. There was no data available regarding Rentrop collateral grading and/or myocardial viability or ischemia inducibility. The potentially significant coronary lesions were not evaluated by FFR (as defined in the Methods section). There were five patients with potentially significant lesions during ablation (however none with previous documented ischemia).
4. Monitoring zones were not uniformly applied below the previously described ICD programmed zones which may influence recurrence rates. However, if patients became symptomatic or if VT was diagnosed during follow-up, ICD zones were reprogrammed accordingly.
5. Long-term mortality endpoints were only based on all-cause mortality (irrespective of cardiovascular vs. non-cardiovascular mortality).
6. There was limited data concerning beta-blocker or amiodarone doses (or HF-directed treatment) prescribed prior to or post-ablation. However, there was no effect caused by post-ablation beta-blocker or amiodarone presence on the evaluated end-points.

## 6. Conclusions

NR-CTO patients treated by RFCA for drug-refractory ES demonstrated a higher ratio of BZ-to-total-scar area. In this analysis, NR-CTO was associated with worse acute procedural results and may as well impact long-term outcomes which should be further assessed in larger patient populations.

## Data Availability

The raw data supporting the conclusions of this article will be made available by the authors, without undue reservation.
