# Supplementary material for: Non-revascularized chronic total occlusions impact on substrate and post-ablation results in drug-refractory electrical storm
Source: Front Cardiovasc Med. 2023 Sep 21;10:1258373. doi: 10.3389/fcvm.2023.1258373 (PMC10552148; doi:10.3389/fcvm.2023.1258373)
Supplement: Supplementary file 1 [file Table1.docx]

Supplementary Table 1. Multivariable models for death and recurrences, respectively, during follow-up in relation to the presence of NR-CTOs. NR-CTO = non-revascularized chronic total occlusion, NYHA = New York Heart Association, SMVT = sustained monomorphic ventricular tachycardia, LVEF = left ventricular ejectionf raction, FMR = functional mitral regurgitation

| Outcome: Death during follow-up | | | |
| --- | --- | --- | --- |
| Multivariable analysis in relation to NR-CTO | | | |
| Model 1 | | | |
| Variable | HR | 95 % CI | p |
| **NR-CTO** | **5.605** | **2.098-14.975** | **0.001** |
| **NYHA III or IV at admission** | **4.616** | **1.686-12.640** | **0.003** |
| Model 2 | | | |
| **NR-CTO** | **3.380** | **1.364-8.372** | **0.009** |
| **Residual SMVT** | **5.147** | **2.003-13.226** | **0.001** |
| Model 3 | | | |
| **NR-CTO** | **2.760** | **1.102-6.912** | **0.03** |
| **Age** | **1.088** | **1.030-1.150** | **0.003** |
| Model 4 | | | |
| **NR-CTO** | **3.278** | **1.299-8.270** | **0.012** |
| LVEF | 0.981 | 0.938-1.025 | 0.388 |
| Model 5 | | | |
| NR-CTO | 2.092 | 0.752-5.821 | 0.15 |
| **Moderate-or-severe FMR** | **3.108** | **1.084-8.914** | **0.03** |
| Outcome: Recurrence during follow-up | | | |
| Multivariable analysis in relation to NR-CTO | | | |
| Variable | HR | 95% CI | p |
| Model 1 | | | |
| NR-CTO | 1.986 | 0.774-5.101 | 0.154 |
| **Residual SMVT** | **9.496** | **3.359-26.849** | **< 0.001** |
| Model 2 | | | |
| **NR-CTO** | **3.114** | **1.194-8.121** | **0.02** |
| **NYHA III or IV** | **3.511** | **1.376-8.961** | **0.009** |
| Model 3 | | | |
| NR-CTO | 2.202 | 0.855-5.672 | 0.102 |
| Age | 1.037 | 0.987-1.089 | 0.15 |
| Model 4 | | | |
| NR-CTO | 2.150 | 0.830-5.568 | 0.11 |
| LVEF | 0.962 | 0.917-1.009 | 0.11 |
| Model 5 | | | |
| NR-CTO | 2.015 | 0.693-5.859 | 0.198 |
| Moderate-or-severe FMR | 1.580 | 0.566-4.415 | 0.383 |

Supplementary Table 2. Supplementary multivariable models for death during follow-up in relation to the presence of residual SMVT at final PVS. NYHA = New York Heart Association, SMVT = sustained monomorphic ventricular tachycardia, LVEF = left ventricular ejectionf raction, FMR = functional mitral regurgitation

| Outcome: death during follow-up | | | |
| --- | --- | --- | --- |
| Variables | HR | 95% CI | p |
| Model 1 | | | |
| **Residual SMVT** | **5.761** | **2.260-14.688** | **<0.001** |
| LVEF | 0.965 | 0.922-1.010 | 0.125 |
| Model 2 | | | |
| **Residual SMVT** | **4.965** | **1.847-11.933** | **<0.001** |
| **Age** | **1.102** | **1.034-1.174** | **0.003** |
| Model 3 | | | |
| **Residual SMVT** | **5.214** | **2.056-13.219** | **0.001** |
| **NYHA III or IV at admission** | **2.675** | **1.059-6.760** | **0.037** |
| Model 4 | | | |
| **Residual SMVT** | **4.990** | **1.974-12.614** | **0.001** |
| **Moderate-or-severe FMR** | **3.968** | **1.538-10.235** | **0.004** |
| Outcome: SMVT recurrence during follow-up | | | |
| Model 1 | | | |
| **Residual SMVT** | **9.532** | **3.417-26.587** | **<0.001** |
| **LVEF** | **0.960** | **0.914-1.008** | **0.099** |
| Model 2 | | | |
| **Residual SMVT** | **9** | **3.179-25.476** | **<0.001** |
| Age | 1.024 | 0.971-1.079 | 0.381 |
| Model 3 | | | |
| **Residual SMVT** | **8.394** | **2.974-23.696** | **<0.001** |
| NYHA III or IV at admission | 1.959 | 0.770-4.985 | 0.158 |
| Model 4 | | | |
| **Residual SMVT** | **9.758** | **3.472-27.426** | **<0.001** |
| Moderate-or-severe FMR | 1.898 | 0.749-4.806 | 0.177 |
